# Crystal structure of (*E*)-1(anthracen-9-ylmethylidene)[2-(morpholin-4-yl)eth­yl]amine

**DOI:** 10.1107/S1600536814018807

**Published:** 2014-08-23

**Authors:** Zeliha Atioğlu, Mehmet Akkurt, Aliasghar Jarrahpour, Mehdi Mohammadi Chermahini, Orhan Büyükgüngör

**Affiliations:** a İlke Education and Health Foundation, Cappadocia Vocational College, The Medical Imaging Techniques Program, 50420 Mustafapaşa, Ürgüp, Nevşehir, Turkey; b Department of Physics, Faculty of Sciences, Erciyes University, 38039 Kayseri, Turkey; c Department of Chemistry, College of Sciences, Shiraz University, 71454 Shiraz, Iran; d Department of Physics, Faculty of Arts and Sciences, Ondokuz Mayıs University, 55139 Samsun, Turkey

**Keywords:** crystal structure, C—H⋯π inter­actions, Schiff bases, anthracene, morpholine, methanimine

## Abstract

The title compound, C_21_H_22_N_2_O, crystallizes with two independent mol­ecules in the asymmetric unit. In both mol­ecules, the anthracene ring systems are almost planar, with maximum deviations of 0.071 (8) and 0.028 (7) Å, and make dihedral angles of 73.4 (2) and 73.3 (2)° with the least-squares planes formed by the four C atoms of the morpholine rings, which adopt a chair conformation. An intra­molecular C—H⋯π inter­action occurs. In the crystal, the packing is stabilized by weak C—H⋯O hydrogen bonds, which connect pairs of molecules into parallel to the *c* axis, and C—H⋯π inter­actions.

## Related literature   

For background to the importance of Schiff bases and their uses, see: Dhar & Taploo (1982 ▶); Witkop & Ramachandran (1964 ▶); Solomon & Lowery (1993 ▶); Gerdemann *et al.* (2002 ▶).
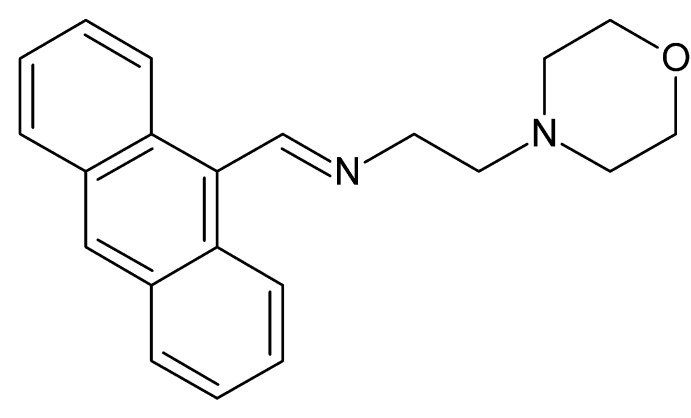


## Experimental   

### Crystal data  

C_21_H_22_N_2_O
*M* *_r_* = 318.41
Monoclinic, 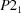

*a* = 6.0451 (3) Å
*b* = 17.8151 (10) Å
*c* = 16.8627 (8) Å
β = 99.690 (4)°
*V* = 1790.10 (16) Å^3^
*Z* = 4
Mo *K*α radiation
μ = 0.07 mm^−1^
*T* = 296 K
0.43 × 0.23 × 0.12 mm

### Data collection  

Stoe IPDS 2 diffractometer
Absorption correction: integration (*X-RED32*; Stoe & Cie, 2002 ▶) *T*_min_ = 0.980, *T*_max_ = 0.993
22689 measured reflections
7325 independent reflections
2245 reflections with *I* > 2σ(*I*)
*R*_int_ = 0.116

### Refinement  

*R*[*F* ^2^ > 2σ(*F* ^2^)] = 0.046
*wR*(*F* ^2^) = 0.076
*S* = 0.71
7325 reflections
433 parameters
1 restraint
H-atom parameters constrained
Δρ_max_ = 0.12 e Å^−3^
Δρ_min_ = −0.10 e Å^−3^

### 

Data collection: *X-AREA* (Stoe & Cie, 2002 ▶); cell refinement: *X-AREA*; data reduction: *X-RED32* (Stoe & Cie, 2002 ▶); program(s) used to solve structure: *SHELXS2013* (Sheldrick, 2008 ▶); program(s) used to refine structure: *SHELXL2013* (Sheldrick, 2008 ▶); molecular graphics: *ORTEP-3 for Windows* (Farrugia, 2012 ▶); software used to prepare material for publication: *WinGX* (Farrugia, 2012 ▶) and *PLATON* (Spek, 2009 ▶).

## Supplementary Material

Crystal structure: contains datablock(s) global, I. DOI: 10.1107/S1600536814018807/xu5815sup1.cif

Structure factors: contains datablock(s) I. DOI: 10.1107/S1600536814018807/xu5815Isup2.hkl

Supporting information file. DOI: 10.1107/S1600536814018807/xu5815Isup3.cml

**Fig. 1.**
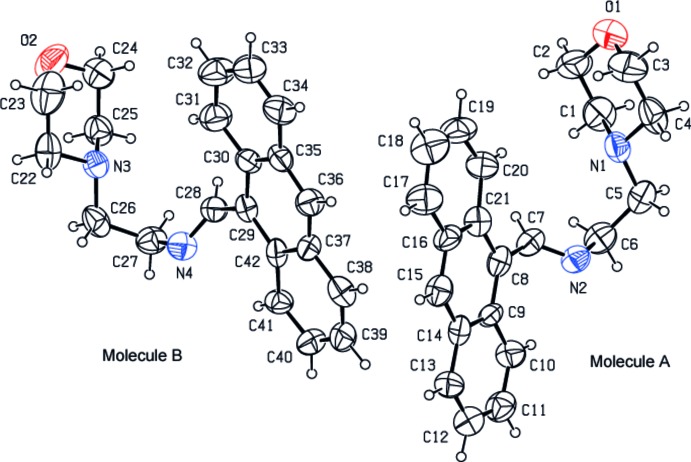
View of the two molecules (A, B) of the title compound in the asymmetric unit with the atom-labelling scheme and 30% probability displacement ellipsoids.

**Fig. 2.**
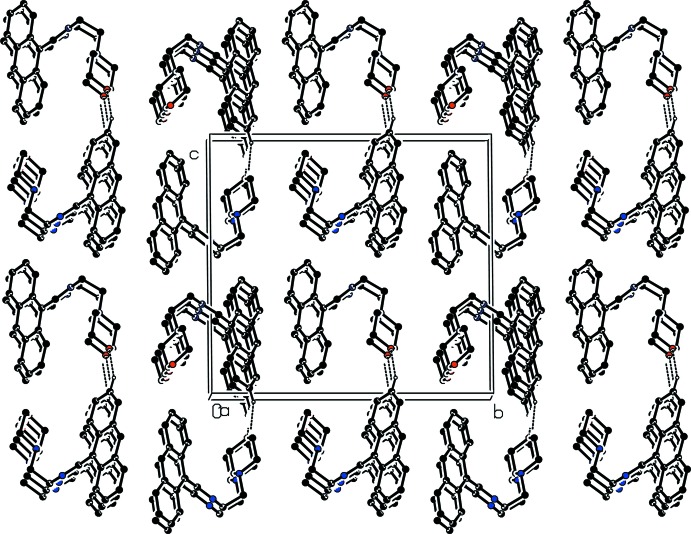
Packing diagram of the title compound viewed down the a axis. Hydrogen bonds are indicated by broken lines. H atoms not participating in hydrogen bonding have been omitted for clarity.

. DOI: 10.1107/S1600536814018807/xu5815fig1.tif
View of the two mol­ecules (A, B) of the title compound in the asymmetric unit with the atom-labelling scheme and 30% probability displacement ellipsoids.

a . DOI: 10.1107/S1600536814018807/xu5815fig2.tif
Packing diagram of the title compound viewed down the *a* axis. Hydrogen bonds are indicated by broken lines. H atoms not participating in hydrogen bonding have been omitted for clarity.

CCDC reference: 1020122

Additional supporting information:  crystallographic information; 3D view; checkCIF report

## Figures and Tables

**Table 1:** Hydrogen-bond geometry (Å, °) *Cg*2 and *Cg*11 are the centroids of the C8/C9/C14–C16/C21 and C37–C42 rings, respectively.

*D*—H⋯*A*	*D*—H	H⋯*A*	*D*⋯*A*	*D*—H⋯*A*
C33—H33⋯O1^i^	0.93	2.59	3.344 (12)	138
C13—H13⋯*Cg*11^ii^	0.93	2.73	3.567 (7)	150
C38—H38⋯*Cg*2	0.93	2.79	3.548 (6)	139

Symmetry codes: (i) 
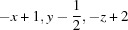
; (ii) 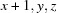.

## supplementary crystallographic information

### S1. Comment

Schiff bases are usually formed by the condensation of a primary amine with an active carbonyl. They are used as pigments and dyes, catalysts, intermediates in organic synthesis, and as polymer stabilisers (Dhar & Taploo, 1982). Schiff bases form an important class of organic compounds with a wide variety of biological properties (Witkop & Ramachandran, 1964). Many studies have been reported regarding the biological activities of Schiff bases, including their anticancer (Solomon & Lowery, 1993), antibacterial (Gerdemann *et al.*, 2002), antifungal, and herbicidal activities. Therefore, Schiff base (I) was synthesized and its X-ray structure is reported here.

As shown in Fig. 1, the asymmetric unit of the title compound (I) consists of two independent molecules (A, B). The anthracene ring systems (C8–C21 and C29–C42) of the both molecules (A, B) are almostly planar [maximum deviations = 0.069 (9) Å for C19 and 0.071 (8) Å for C12 in molecule A, and 0.028 (7) Å for C39 and C30 in molecule B]. They make dihedral angles of 73.4 (2) and 73.3 (2)°, respectively, with the least-squares planes formed by the four C atoms of the morpholine rings (C1–C4/N1/O1 and C22–C25/N3/O2), which adopts a chair conformation. The puckering parameters of the morpholine rings are Q_T_ = 0.575 (9) Å, θ = 1.4 (9) °, φ = 142 (64) ° for molecule A, and Q_T_ = 0.569 (8) Å, θ = 0.0 (8) °, φ = 223 (34) ° for molecule B, respectively.

The (C5–C6–N2–C7 and C26–C27–N4–C28) torsion angles of the bridge –C–C–N–C– groups is -103.0 (7) ° for molecule A and 101.1 (6)° for molecule B. The bond lengths and angles in both molecules (A, B) may be regarded as normal, and they are similar with each other.

In the crystal structure, weak C—H···O hydrogen bonds (Table 1, Fig. 2) which connect pairs of molecules into parallel to the *c* axis, further stabilize the packing supported by C–H···π interactions (Table 1).

### S2. Experimental

Reaction of anthracene-9-carbaldehyde (1.00 mmol) with 2-morpholinoethanamine (1.00 mmol) in refluxing ethanol gave the title compound (I). Recrystallization from ethanol gave yellow crystals in 85% yield. Mp: 381–383 K. IR (KBr) cm^-1^:1643 (C=N). ^1^H-NMR (250 MHz, CDCl_3_), δ (p.p.m.): 2.58 (CH_2_—N morpholine, t, 4H, J=5 Hz), 2.84 (morpholine-CH_2_—CH_2_, t, 2H, J=7.5 Hz), 3.73 (CH_2_—O morpholine, t, 4H, J=5 Hz), 4.00 (morpholine-CH_2_—CH_2_, t, 2H, J=5 Hz), 7.37–7.94 (aromatic H, m, 9H), 8.45 (HC=N, s, 1H). ^13^CNMR (62.9 MHz, CDCl_3_), δ (p.p.m): 53.9 (CH_2_—N morpholine), 58.9 and 59.6 (N—CH_2_—CH_2_—N), 67.0 (CH_2_—O morpholin), 124.9–131.2 (aromatic carbons), 161.4 (C=N).

### S3. Refinement

H atoms were located geometrically with C—H = 0.93 and 0.97 Å, and refined using a riding model with *U*_iso_(H) = 1.2*U*_eq_(C) for the aromatic and methylene H atoms. The crystal quality and data was not good enough so a sufficient fraction of the unique data is above the 2 sigma level. A total of 749 estimated Friedel pairs were merged before refinement and not used as independent data. The Flack parameter was found to be meaningless and was omitted.

### Crystal data

**Table:**

C_21_H_22_N_2_O	*F*(000) = 680
*M**_r_* = 318.41	*D*_x_ = 1.181 Mg m^−^^3^
Monoclinic, *P*2_1_	Mo *K*α radiation, λ = 0.71073 Å
Hall symbol: P 2yb	Cell parameters from 10759 reflections
*a* = 6.0451 (3) Å	θ = 1.7–27.1°
*b* = 17.8151 (10) Å	µ = 0.07 mm^−^^1^
*c* = 16.8627 (8) Å	*T* = 296 K
β = 99.690 (4)°	Needle, yellow
*V* = 1790.10 (16) Å^3^	0.43 × 0.23 × 0.12 mm
*Z* = 4	

### Data collection

**Table:**

Stoe IPDS 2 diffractometer	7325 independent reflections
Radiation source: sealed X-ray tube, 12 x 0.4 mm long-fine focus	2245 reflections with *I* > 2σ(*I*)
Plane graphite monochromator	*R*_int_ = 0.116
Detector resolution: 6.67 pixels mm^-1^	θ_max_ = 26.5°, θ_min_ = 1.7°
ω scans	*h* = −7→7
Absorption correction: integration (*X-RED32*; Stoe & Cie, 2002)	*k* = −22→22
*T*_min_ = 0.980, *T*_max_ = 0.993	*l* = −21→21
22689 measured reflections	

### Refinement

**Table:**

Refinement on *F*^2^	1 restraint
Least-squares matrix: full	Hydrogen site location: inferred from neighbouring sites
*R*[*F*^2^ > 2σ(*F*^2^)] = 0.046	H-atom parameters constrained
*wR*(*F*^2^) = 0.076	*w* = 1/[σ^2^(*F*_o_^2^) + (0.0001*P*)^2^] where *P* = (*F*_o_^2^ + 2*F*_c_^2^)/3
*S* = 0.71	(Δ/σ)_max_ = 0.001
7325 reflections	Δρ_max_ = 0.12 e Å^−^^3^
433 parameters	Δρ_min_ = −0.10 e Å^−^^3^

### Special details

**Table:**

Geometry. Bond distances, angles *etc*. have been calculated using the rounded fractional coordinates. All su's are estimated from the variances of the (full) variance-covariance matrix. The cell e.s.d.'s are taken into account in the estimation of distances, angles and torsion angles
Refinement. Refinement on *F*^2^ for ALL reflections except those flagged by the user for potential systematic errors. Weighted *R*-factors *wR* and all goodnesses of fit *S* are based on *F*^2^, conventional *R*-factors *R* are based on *F*, with *F* set to zero for negative *F*^2^. The observed criterion of *F*^2^ > σ(*F*^2^) is used only for calculating -*R*-factor-obs *etc*. and is not relevant to the choice of reflections for refinement. *R*-factors based on *F*^2^ are statistically about twice as large as those based on *F*, and *R*-factors based on ALL data will be even larger.

### Fractional atomic coordinates and isotropic or equivalent isotropic displacement parameters (Å^2^)

**Table:**

	*x*	*y*	*z*	*U*_iso_*/*U*_eq_	
O1	0.5030 (14)	1.1304 (3)	0.8402 (4)	0.151 (3)	
N1	0.6844 (9)	1.0984 (3)	0.6979 (4)	0.091 (3)	
N2	0.9321 (9)	1.0010 (3)	0.5933 (3)	0.083 (2)	
C1	0.4497 (12)	1.0827 (4)	0.7044 (5)	0.125 (4)	
C2	0.4292 (17)	1.0681 (5)	0.7902 (6)	0.165 (5)	
C3	0.7314 (19)	1.1459 (5)	0.8339 (6)	0.155 (6)	
C4	0.7548 (14)	1.1610 (3)	0.7501 (6)	0.121 (4)	
C5	0.7151 (11)	1.1119 (3)	0.6140 (4)	0.102 (3)	
C6	0.7207 (12)	1.0390 (4)	0.5671 (4)	0.103 (3)	
C7	0.9286 (11)	0.9462 (4)	0.6392 (4)	0.083 (3)	
C8	1.1260 (11)	0.9000 (3)	0.6682 (5)	0.075 (3)	
C9	1.2703 (12)	0.8691 (3)	0.6193 (4)	0.069 (3)	
C10	1.2220 (11)	0.8797 (3)	0.5362 (5)	0.095 (3)	
C11	1.3547 (14)	0.8477 (4)	0.4871 (4)	0.103 (3)	
C12	1.5368 (14)	0.8018 (4)	0.5200 (5)	0.112 (4)	
C13	1.5893 (12)	0.7909 (3)	0.5997 (5)	0.094 (3)	
C14	1.4558 (13)	0.8241 (3)	0.6516 (5)	0.079 (3)	
C15	1.4988 (12)	0.8134 (4)	0.7345 (5)	0.089 (3)	
C16	1.3580 (16)	0.8435 (4)	0.7827 (4)	0.089 (4)	
C17	1.4078 (14)	0.8284 (4)	0.8665 (6)	0.119 (4)	
C18	1.2768 (16)	0.8546 (5)	0.9180 (5)	0.131 (5)	
C19	1.0911 (18)	0.8962 (5)	0.8894 (6)	0.120 (4)	
C20	1.0344 (12)	0.9135 (4)	0.8089 (5)	0.097 (4)	
C21	1.1684 (13)	0.8875 (4)	0.7520 (5)	0.082 (3)	
O2	−0.0218 (13)	0.3492 (4)	0.9153 (4)	0.143 (3)	
N3	0.1892 (9)	0.3720 (2)	0.7801 (3)	0.075 (2)	
N4	0.4442 (8)	0.4611 (3)	0.6707 (3)	0.086 (2)	
C22	0.2622 (11)	0.3138 (3)	0.8388 (5)	0.104 (3)	
C23	0.2071 (17)	0.3359 (5)	0.9198 (5)	0.147 (5)	
C24	−0.0968 (13)	0.4068 (4)	0.8585 (5)	0.122 (4)	
C25	−0.0489 (11)	0.3848 (3)	0.7777 (4)	0.094 (3)	
C26	0.2383 (11)	0.3506 (3)	0.6997 (5)	0.096 (3)	
C27	0.2407 (11)	0.4180 (3)	0.6453 (4)	0.091 (3)	
C28	0.4249 (10)	0.5193 (3)	0.7114 (4)	0.078 (3)	
C29	0.6164 (11)	0.5675 (3)	0.7442 (4)	0.071 (3)	
C30	0.6443 (13)	0.5896 (3)	0.8261 (4)	0.075 (3)	
C31	0.4994 (12)	0.5602 (4)	0.8772 (5)	0.105 (4)	
C32	0.5272 (15)	0.5804 (5)	0.9561 (5)	0.122 (4)	
C33	0.7021 (18)	0.6295 (4)	0.9876 (5)	0.123 (4)	
C34	0.8488 (14)	0.6564 (4)	0.9425 (5)	0.110 (4)	
C35	0.8258 (13)	0.6379 (3)	0.8593 (5)	0.079 (3)	
C36	0.9658 (13)	0.6628 (3)	0.8079 (5)	0.087 (3)	
C37	0.9342 (13)	0.6426 (4)	0.7280 (5)	0.079 (3)	
C38	1.0851 (13)	0.6725 (3)	0.6783 (5)	0.098 (4)	
C39	1.0621 (14)	0.6555 (4)	0.5994 (5)	0.096 (4)	
C40	0.8943 (14)	0.6070 (4)	0.5655 (5)	0.099 (3)	
C41	0.7469 (11)	0.5766 (3)	0.6099 (4)	0.076 (3)	
C42	0.7638 (11)	0.5937 (3)	0.6937 (4)	0.070 (3)	
H1A	0.39760	1.03920	0.67190	0.1490*	
H1B	0.35650	1.12520	0.68430	0.1490*	
H2A	0.27380	1.05740	0.79360	0.1980*	
H2B	0.51790	1.02440	0.80930	0.1980*	
H3A	0.82430	1.10330	0.85390	0.1850*	
H3B	0.78320	1.18910	0.86690	0.1850*	
H4A	0.66560	1.20470	0.73100	0.1450*	
H4B	0.91050	1.17250	0.74780	0.1450*	
H5A	0.85430	1.13900	0.61410	0.1220*	
H5B	0.59320	1.14300	0.58740	0.1220*	
H6A	0.59760	1.00690	0.57590	0.1230*	
H6B	0.70340	1.04990	0.51000	0.1230*	
H7	0.79390	0.93400	0.65570	0.0990*	
H10	1.09900	0.90860	0.51400	0.1140*	
H11	1.32440	0.85630	0.43200	0.1230*	
H12	1.62200	0.77870	0.48600	0.1340*	
H13	1.71280	0.76170	0.62070	0.1130*	
H15	1.62360	0.78570	0.75760	0.1080*	
H17	1.53360	0.79990	0.88650	0.1420*	
H18	1.31310	0.84420	0.97270	0.1570*	
H19	1.00060	0.91330	0.92510	0.1440*	
H20	0.90770	0.94250	0.79130	0.1160*	
H22A	0.42270	0.30640	0.84310	0.1250*	
H22B	0.18780	0.26700	0.82130	0.1250*	
H23A	0.25330	0.29600	0.95820	0.1760*	
H23B	0.29020	0.38080	0.93880	0.1760*	
H24A	−0.02060	0.45340	0.87550	0.1470*	
H24B	−0.25680	0.41450	0.85570	0.1470*	
H25A	−0.13120	0.33940	0.75990	0.1130*	
H25B	−0.09950	0.42420	0.73920	0.1130*	
H26A	0.12550	0.31530	0.67470	0.1150*	
H26B	0.38300	0.32580	0.70620	0.1150*	
H27A	0.23450	0.40150	0.59010	0.1090*	
H27B	0.11050	0.44920	0.64770	0.1090*	
H28	0.28310	0.53250	0.72120	0.0930*	
H31	0.38550	0.52700	0.85650	0.1270*	
H32	0.43080	0.56190	0.98900	0.1460*	
H33	0.71720	0.64390	1.04130	0.1480*	
H34	0.96590	0.68720	0.96600	0.1330*	
H36	1.08510	0.69420	0.82800	0.1040*	
H38	1.20080	0.70420	0.70110	0.1170*	
H39	1.15880	0.67640	0.56800	0.1150*	
H40	0.88130	0.59470	0.51130	0.1190*	
H41	0.63450	0.54450	0.58540	0.0910*	

### Atomic displacement parameters (Å^2^)

**Table:**

	*U*^11^	*U*^22^	*U*^33^	*U*^12^	*U*^13^	*U*^23^
O1	0.189 (7)	0.123 (4)	0.161 (6)	0.003 (5)	0.085 (5)	−0.019 (4)
N1	0.086 (5)	0.067 (3)	0.122 (5)	−0.005 (3)	0.022 (4)	−0.006 (3)
N2	0.075 (4)	0.098 (4)	0.077 (4)	0.005 (3)	0.012 (3)	0.012 (3)
C1	0.085 (6)	0.114 (5)	0.184 (9)	−0.032 (4)	0.049 (7)	−0.019 (5)
C2	0.210 (11)	0.141 (8)	0.178 (9)	−0.054 (7)	0.127 (9)	−0.034 (7)
C3	0.194 (13)	0.161 (9)	0.115 (9)	−0.002 (8)	0.044 (9)	−0.033 (6)
C4	0.113 (7)	0.075 (5)	0.171 (9)	−0.012 (4)	0.017 (6)	−0.023 (5)
C5	0.093 (6)	0.102 (5)	0.110 (6)	−0.004 (4)	0.018 (5)	0.029 (5)
C6	0.101 (6)	0.124 (5)	0.079 (5)	0.002 (5)	0.001 (5)	0.015 (4)
C7	0.083 (5)	0.096 (5)	0.070 (5)	−0.019 (4)	0.014 (4)	0.007 (4)
C8	0.060 (5)	0.075 (4)	0.086 (6)	−0.024 (4)	−0.002 (5)	0.011 (4)
C9	0.082 (5)	0.073 (4)	0.056 (5)	−0.018 (4)	0.023 (4)	0.004 (4)
C10	0.095 (6)	0.097 (5)	0.098 (6)	−0.001 (4)	0.030 (5)	−0.003 (4)
C11	0.120 (7)	0.109 (6)	0.076 (5)	−0.010 (5)	0.009 (5)	0.008 (4)
C12	0.127 (8)	0.105 (5)	0.112 (7)	−0.001 (5)	0.044 (6)	0.004 (5)
C13	0.092 (6)	0.092 (5)	0.105 (6)	0.008 (4)	0.037 (6)	−0.004 (5)
C14	0.081 (6)	0.062 (4)	0.095 (6)	−0.004 (4)	0.014 (5)	0.019 (4)
C15	0.090 (6)	0.090 (5)	0.082 (6)	−0.007 (4)	−0.002 (5)	0.012 (5)
C16	0.122 (8)	0.093 (5)	0.054 (5)	−0.027 (5)	0.021 (5)	−0.005 (4)
C17	0.100 (7)	0.146 (7)	0.111 (8)	−0.003 (5)	0.021 (6)	−0.011 (6)
C18	0.110 (8)	0.175 (9)	0.097 (7)	0.017 (6)	−0.011 (6)	0.020 (6)
C19	0.145 (9)	0.152 (8)	0.068 (6)	−0.016 (6)	0.034 (6)	−0.020 (5)
C20	0.084 (6)	0.111 (6)	0.097 (7)	0.007 (4)	0.021 (5)	−0.007 (5)
C21	0.070 (5)	0.082 (5)	0.088 (6)	−0.005 (4)	0.000 (5)	0.001 (4)
O2	0.181 (6)	0.148 (5)	0.107 (5)	−0.012 (5)	0.042 (5)	0.041 (4)
N3	0.068 (4)	0.072 (3)	0.083 (4)	0.010 (3)	0.006 (3)	0.003 (3)
N4	0.076 (4)	0.094 (3)	0.086 (4)	−0.010 (3)	0.009 (3)	−0.013 (3)
C22	0.088 (6)	0.079 (4)	0.137 (7)	−0.004 (4)	−0.007 (6)	0.021 (5)
C23	0.159 (10)	0.148 (7)	0.113 (8)	−0.007 (8)	−0.037 (8)	0.040 (6)
C24	0.134 (8)	0.104 (6)	0.138 (8)	0.000 (5)	0.049 (6)	−0.010 (6)
C25	0.085 (6)	0.086 (5)	0.112 (6)	0.005 (4)	0.018 (5)	0.001 (4)
C26	0.084 (5)	0.071 (4)	0.133 (7)	−0.015 (4)	0.016 (5)	−0.028 (4)
C27	0.091 (6)	0.105 (5)	0.078 (5)	−0.011 (4)	0.014 (4)	−0.011 (4)
C28	0.079 (5)	0.078 (4)	0.078 (5)	−0.001 (4)	0.020 (4)	−0.004 (4)
C29	0.075 (5)	0.050 (3)	0.086 (6)	−0.001 (3)	0.009 (5)	−0.003 (4)
C30	0.089 (6)	0.067 (4)	0.070 (5)	0.012 (4)	0.018 (5)	−0.007 (4)
C31	0.116 (7)	0.115 (6)	0.088 (6)	−0.005 (5)	0.025 (6)	−0.004 (5)
C32	0.136 (8)	0.152 (8)	0.082 (7)	−0.023 (6)	0.029 (6)	−0.023 (5)
C33	0.184 (10)	0.117 (6)	0.072 (6)	0.002 (6)	0.032 (6)	−0.009 (5)
C34	0.145 (8)	0.106 (5)	0.077 (6)	−0.010 (5)	0.010 (5)	−0.033 (4)
C35	0.093 (6)	0.062 (4)	0.082 (6)	0.004 (4)	0.011 (5)	−0.011 (4)
C36	0.087 (6)	0.089 (5)	0.084 (6)	−0.010 (4)	0.016 (5)	−0.008 (4)
C37	0.088 (6)	0.068 (4)	0.088 (6)	−0.002 (4)	0.032 (5)	0.012 (4)
C38	0.105 (7)	0.074 (4)	0.112 (7)	0.003 (4)	0.014 (6)	−0.015 (5)
C39	0.096 (6)	0.078 (5)	0.120 (8)	−0.013 (4)	0.037 (6)	0.001 (4)
C40	0.108 (6)	0.100 (5)	0.101 (6)	0.002 (5)	0.050 (6)	0.012 (5)
C41	0.082 (5)	0.086 (4)	0.059 (5)	0.010 (3)	0.010 (4)	−0.003 (3)
C42	0.066 (5)	0.048 (3)	0.094 (6)	−0.003 (3)	0.009 (5)	0.006 (4)

### Geometric parameters (Å, º)

**Table:**

O1—C2	1.420 (11)	C12—H12	0.9300
O1—C3	1.429 (14)	C13—H13	0.9300
O2—C23	1.393 (13)	C15—H15	0.9300
O2—C24	1.425 (10)	C17—H17	0.9300
N1—C1	1.468 (9)	C18—H18	0.9300
N1—C5	1.477 (9)	C19—H19	0.9300
N1—C4	1.440 (10)	C20—H20	0.9300
N2—C7	1.248 (9)	C22—C23	1.512 (12)
N2—C6	1.448 (9)	C24—C25	1.492 (11)
N3—C26	1.486 (9)	C26—C27	1.513 (9)
N3—C22	1.450 (8)	C28—C29	1.472 (9)
N3—C25	1.451 (9)	C29—C30	1.419 (9)
N4—C28	1.260 (8)	C29—C42	1.412 (9)
N4—C27	1.452 (8)	C30—C31	1.428 (11)
C1—C2	1.496 (13)	C30—C35	1.432 (10)
C3—C4	1.469 (14)	C31—C32	1.361 (12)
C5—C6	1.524 (9)	C32—C33	1.406 (13)
C7—C8	1.464 (10)	C33—C34	1.350 (13)
C8—C9	1.409 (10)	C34—C35	1.425 (12)
C8—C21	1.411 (12)	C35—C36	1.383 (11)
C9—C10	1.395 (11)	C36—C37	1.377 (12)
C9—C14	1.411 (10)	C37—C38	1.441 (11)
C10—C11	1.370 (11)	C37—C42	1.398 (10)
C11—C12	1.408 (11)	C38—C39	1.349 (12)
C12—C13	1.342 (12)	C39—C40	1.381 (11)
C13—C14	1.416 (11)	C40—C41	1.368 (11)
C14—C15	1.391 (12)	C41—C42	1.432 (9)
C15—C16	1.380 (11)	C22—H22A	0.9700
C16—C21	1.413 (12)	C22—H22B	0.9700
C16—C17	1.420 (12)	C23—H23A	0.9700
C17—C18	1.353 (13)	C23—H23B	0.9700
C18—C19	1.364 (14)	C24—H24A	0.9700
C19—C20	1.378 (13)	C24—H24B	0.9700
C20—C21	1.433 (11)	C25—H25A	0.9700
C1—H1A	0.9700	C25—H25B	0.9700
C1—H1B	0.9700	C26—H26A	0.9700
C2—H2A	0.9700	C26—H26B	0.9700
C2—H2B	0.9700	C27—H27A	0.9700
C3—H3A	0.9700	C27—H27B	0.9700
C3—H3B	0.9700	C28—H28	0.9300
C4—H4A	0.9700	C31—H31	0.9300
C4—H4B	0.9700	C32—H32	0.9300
C5—H5B	0.9700	C33—H33	0.9300
C5—H5A	0.9700	C34—H34	0.9300
C6—H6A	0.9700	C36—H36	0.9300
C6—H6B	0.9700	C38—H38	0.9300
C7—H7	0.9300	C39—H39	0.9300
C10—H10	0.9300	C40—H40	0.9300
C11—H11	0.9300	C41—H41	0.9300
			
C2—O1—C3	108.5 (7)	C19—C18—H18	120.00
C23—O2—C24	111.1 (7)	C20—C19—H19	119.00
C1—N1—C5	112.4 (6)	C18—C19—H19	119.00
C4—N1—C5	112.8 (5)	C21—C20—H20	120.00
C1—N1—C4	107.0 (6)	C19—C20—H20	120.00
C6—N2—C7	116.7 (6)	N3—C22—C23	110.0 (5)
C22—N3—C25	108.5 (5)	O2—C23—C22	111.3 (7)
C22—N3—C26	110.8 (5)	O2—C24—C25	109.6 (6)
C25—N3—C26	111.4 (5)	N3—C25—C24	111.1 (6)
C27—N4—C28	116.4 (5)	N3—C26—C27	111.9 (5)
N1—C1—C2	110.1 (7)	N4—C27—C26	109.6 (5)
O1—C2—C1	111.7 (7)	N4—C28—C29	123.1 (6)
O1—C3—C4	111.0 (8)	C28—C29—C30	119.0 (6)
N1—C4—C3	112.3 (6)	C28—C29—C42	120.2 (6)
N1—C5—C6	112.1 (5)	C30—C29—C42	120.8 (6)
N2—C6—C5	109.6 (5)	C29—C30—C31	120.1 (6)
N2—C7—C8	123.3 (6)	C29—C30—C35	120.3 (7)
C9—C8—C21	119.9 (6)	C31—C30—C35	119.5 (6)
C7—C8—C21	115.1 (6)	C30—C31—C32	120.4 (7)
C7—C8—C9	125.0 (7)	C31—C32—C33	119.7 (8)
C8—C9—C10	119.3 (6)	C32—C33—C34	122.1 (8)
C10—C9—C14	118.9 (6)	C33—C34—C35	120.5 (7)
C8—C9—C14	121.7 (7)	C30—C35—C34	117.7 (7)
C9—C10—C11	120.4 (6)	C30—C35—C36	117.2 (7)
C10—C11—C12	120.1 (7)	C34—C35—C36	125.1 (7)
C11—C12—C13	121.1 (7)	C35—C36—C37	122.1 (7)
C12—C13—C14	119.7 (7)	C36—C37—C38	118.4 (7)
C9—C14—C13	119.8 (7)	C36—C37—C42	122.6 (7)
C13—C14—C15	122.3 (7)	C38—C37—C42	119.0 (7)
C9—C14—C15	117.9 (7)	C37—C38—C39	121.4 (7)
C14—C15—C16	120.6 (7)	C38—C39—C40	119.7 (8)
C15—C16—C21	122.8 (7)	C39—C40—C41	121.5 (7)
C17—C16—C21	119.5 (8)	C40—C41—C42	120.7 (6)
C15—C16—C17	117.6 (8)	C29—C42—C37	116.9 (6)
C16—C17—C18	121.6 (8)	C29—C42—C41	125.3 (6)
C17—C18—C19	119.8 (8)	C37—C42—C41	117.8 (6)
C18—C19—C20	121.6 (9)	N3—C22—H22A	110.00
C19—C20—C21	120.8 (8)	N3—C22—H22B	110.00
C8—C21—C20	126.3 (7)	C23—C22—H22A	110.00
C16—C21—C20	116.7 (7)	C23—C22—H22B	110.00
C8—C21—C16	117.0 (7)	H22A—C22—H22B	108.00
C2—C1—H1A	110.00	O2—C23—H23A	109.00
C2—C1—H1B	110.00	O2—C23—H23B	109.00
N1—C1—H1A	110.00	C22—C23—H23A	109.00
N1—C1—H1B	110.00	C22—C23—H23B	109.00
H1A—C1—H1B	108.00	H23A—C23—H23B	108.00
O1—C2—H2A	109.00	O2—C24—H24A	110.00
O1—C2—H2B	109.00	O2—C24—H24B	110.00
H2A—C2—H2B	108.00	C25—C24—H24A	110.00
C1—C2—H2B	109.00	C25—C24—H24B	110.00
C1—C2—H2A	109.00	H24A—C24—H24B	108.00
O1—C3—H3B	109.00	N3—C25—H25A	109.00
O1—C3—H3A	109.00	N3—C25—H25B	109.00
C4—C3—H3B	109.00	C24—C25—H25A	109.00
H3A—C3—H3B	108.00	C24—C25—H25B	109.00
C4—C3—H3A	110.00	H25A—C25—H25B	108.00
C3—C4—H4A	109.00	N3—C26—H26A	109.00
C3—C4—H4B	109.00	N3—C26—H26B	109.00
N1—C4—H4A	109.00	C27—C26—H26A	109.00
N1—C4—H4B	109.00	C27—C26—H26B	109.00
H4A—C4—H4B	108.00	H26A—C26—H26B	108.00
H5A—C5—H5B	108.00	N4—C27—H27A	110.00
N1—C5—H5A	109.00	N4—C27—H27B	110.00
N1—C5—H5B	109.00	C26—C27—H27A	110.00
C6—C5—H5B	109.00	C26—C27—H27B	110.00
C6—C5—H5A	109.00	H27A—C27—H27B	108.00
N2—C6—H6B	110.00	N4—C28—H28	119.00
C5—C6—H6A	110.00	C29—C28—H28	118.00
N2—C6—H6A	110.00	C30—C31—H31	120.00
H6A—C6—H6B	108.00	C32—C31—H31	120.00
C5—C6—H6B	110.00	C31—C32—H32	120.00
N2—C7—H7	118.00	C33—C32—H32	120.00
C8—C7—H7	118.00	C32—C33—H33	119.00
C11—C10—H10	120.00	C34—C33—H33	119.00
C9—C10—H10	120.00	C33—C34—H34	120.00
C10—C11—H11	120.00	C35—C34—H34	120.00
C12—C11—H11	120.00	C35—C36—H36	119.00
C11—C12—H12	119.00	C37—C36—H36	119.00
C13—C12—H12	119.00	C37—C38—H38	119.00
C14—C13—H13	120.00	C39—C38—H38	119.00
C12—C13—H13	120.00	C38—C39—H39	120.00
C16—C15—H15	120.00	C40—C39—H39	120.00
C14—C15—H15	120.00	C39—C40—H40	119.00
C16—C17—H17	119.00	C41—C40—H40	119.00
C18—C17—H17	119.00	C40—C41—H41	120.00
C17—C18—H18	120.00	C42—C41—H41	120.00
			
C3—O1—C2—C1	−58.4 (9)	C15—C16—C21—C8	0.1 (11)
C2—O1—C3—C4	57.9 (9)	C15—C16—C17—C18	−178.9 (8)
C23—O2—C24—C25	58.5 (8)	C17—C16—C21—C8	−179.4 (7)
C24—O2—C23—C22	−58.3 (9)	C21—C16—C17—C18	0.6 (12)
C4—N1—C5—C6	159.8 (6)	C15—C16—C21—C20	178.5 (7)
C5—N1—C1—C2	179.0 (6)	C16—C17—C18—C19	0.4 (13)
C1—N1—C5—C6	−79.2 (7)	C17—C18—C19—C20	−1.1 (14)
C4—N1—C1—C2	−56.7 (8)	C18—C19—C20—C21	0.7 (13)
C1—N1—C4—C3	58.0 (9)	C19—C20—C21—C16	0.4 (11)
C5—N1—C4—C3	−177.9 (7)	C19—C20—C21—C8	178.6 (8)
C6—N2—C7—C8	177.3 (6)	N3—C22—C23—O2	57.7 (8)
C7—N2—C6—C5	103.0 (7)	O2—C24—C25—N3	−58.8 (7)
C26—N3—C25—C24	−179.6 (5)	N3—C26—C27—N4	74.0 (7)
C26—N3—C22—C23	−179.0 (6)	N4—C28—C29—C30	−131.7 (7)
C25—N3—C26—C27	78.9 (6)	N4—C28—C29—C42	51.0 (9)
C25—N3—C22—C23	−56.4 (7)	C28—C29—C30—C31	5.2 (9)
C22—N3—C26—C27	−160.2 (5)	C28—C29—C30—C35	−178.5 (6)
C22—N3—C25—C24	58.2 (6)	C42—C29—C30—C31	−177.5 (6)
C28—N4—C27—C26	−101.1 (6)	C42—C29—C30—C35	−1.2 (9)
C27—N4—C28—C29	177.7 (6)	C28—C29—C42—C37	176.5 (6)
N1—C1—C2—O1	59.5 (9)	C28—C29—C42—C41	−0.9 (9)
O1—C3—C4—N1	−60.2 (9)	C30—C29—C42—C37	−0.8 (9)
N1—C5—C6—N2	−73.5 (7)	C30—C29—C42—C41	−178.2 (6)
N2—C7—C8—C9	−49.0 (10)	C29—C30—C31—C32	179.1 (7)
N2—C7—C8—C21	130.6 (7)	C35—C30—C31—C32	2.8 (11)
C21—C8—C9—C10	177.8 (6)	C29—C30—C35—C34	−178.2 (6)
C7—C8—C9—C14	−179.0 (6)	C29—C30—C35—C36	1.6 (9)
C7—C8—C21—C20	2.1 (10)	C31—C30—C35—C34	−2.0 (9)
C7—C8—C21—C16	−179.7 (6)	C31—C30—C35—C36	177.8 (6)
C9—C8—C21—C20	−178.3 (7)	C30—C31—C32—C33	−1.1 (12)
C7—C8—C9—C10	−2.6 (9)	C31—C32—C33—C34	−1.6 (13)
C21—C8—C9—C14	1.4 (9)	C32—C33—C34—C35	2.4 (13)
C9—C8—C21—C16	−0.1 (10)	C33—C34—C35—C30	−0.6 (11)
C10—C9—C14—C15	−179.1 (6)	C33—C34—C35—C36	179.6 (7)
C14—C9—C10—C11	−0.9 (9)	C30—C35—C36—C37	0.1 (10)
C8—C9—C14—C13	176.6 (6)	C34—C35—C36—C37	179.9 (7)
C10—C9—C14—C13	0.2 (9)	C35—C36—C37—C38	178.6 (6)
C8—C9—C10—C11	−177.4 (6)	C35—C36—C37—C42	−2.2 (11)
C8—C9—C14—C15	−2.7 (9)	C36—C37—C38—C39	−179.4 (7)
C9—C10—C11—C12	2.1 (10)	C42—C37—C38—C39	1.4 (10)
C10—C11—C12—C13	−2.7 (11)	C36—C37—C42—C29	2.5 (10)
C11—C12—C13—C14	2.0 (11)	C36—C37—C42—C41	−180.0 (6)
C12—C13—C14—C15	178.5 (7)	C38—C37—C42—C29	−178.3 (6)
C12—C13—C14—C9	−0.8 (10)	C38—C37—C42—C41	−0.8 (9)
C13—C14—C15—C16	−176.6 (7)	C37—C38—C39—C40	−1.6 (11)
C9—C14—C15—C16	2.6 (10)	C38—C39—C40—C41	1.3 (12)
C14—C15—C16—C17	178.0 (7)	C39—C40—C41—C42	−0.7 (11)
C14—C15—C16—C21	−1.4 (12)	C40—C41—C42—C29	177.7 (6)
C17—C16—C21—C20	−0.9 (11)	C40—C41—C42—C37	0.4 (9)

### Hydrogen-bond geometry (Å, º)

Cg2 and Cg11 are the centroids of the C8/C9/C14–C16/C21 and C37–C42 rings, respectively.

**Table:**

*D*—H···*A*	*D*—H	H···*A*	*D*···*A*	*D*—H···*A*
C10—H10···N2	0.93	2.44	3.039 (8)	122
C33—H33···O1^i^	0.93	2.59	3.344 (12)	138
C41—H41···N4	0.93	2.48	3.044 (8)	119
C13—H13···*Cg*11^ii^	0.93	2.73	3.567 (7)	150
C38—H38···*Cg*2	0.93	2.79	3.548 (6)	139

Symmetry codes: (i) −*x*+1, *y*−1/2, −*z*+2; (ii) *x*+1, *y*, *z*.
