# Supplementary figures and images for: Crystal structure of (E)-1(anthracen-9-ylmethylidene)[2-(morpholin-4-yl)eth­yl]amine
Source: Acta Crystallogr Sect E Struct Rep Online. 2014 Aug 23;70(Pt 9):o1045–6. doi: 10.1107/S1600536814018807 (PMC4186061; doi:10.1107/S1600536814018807)

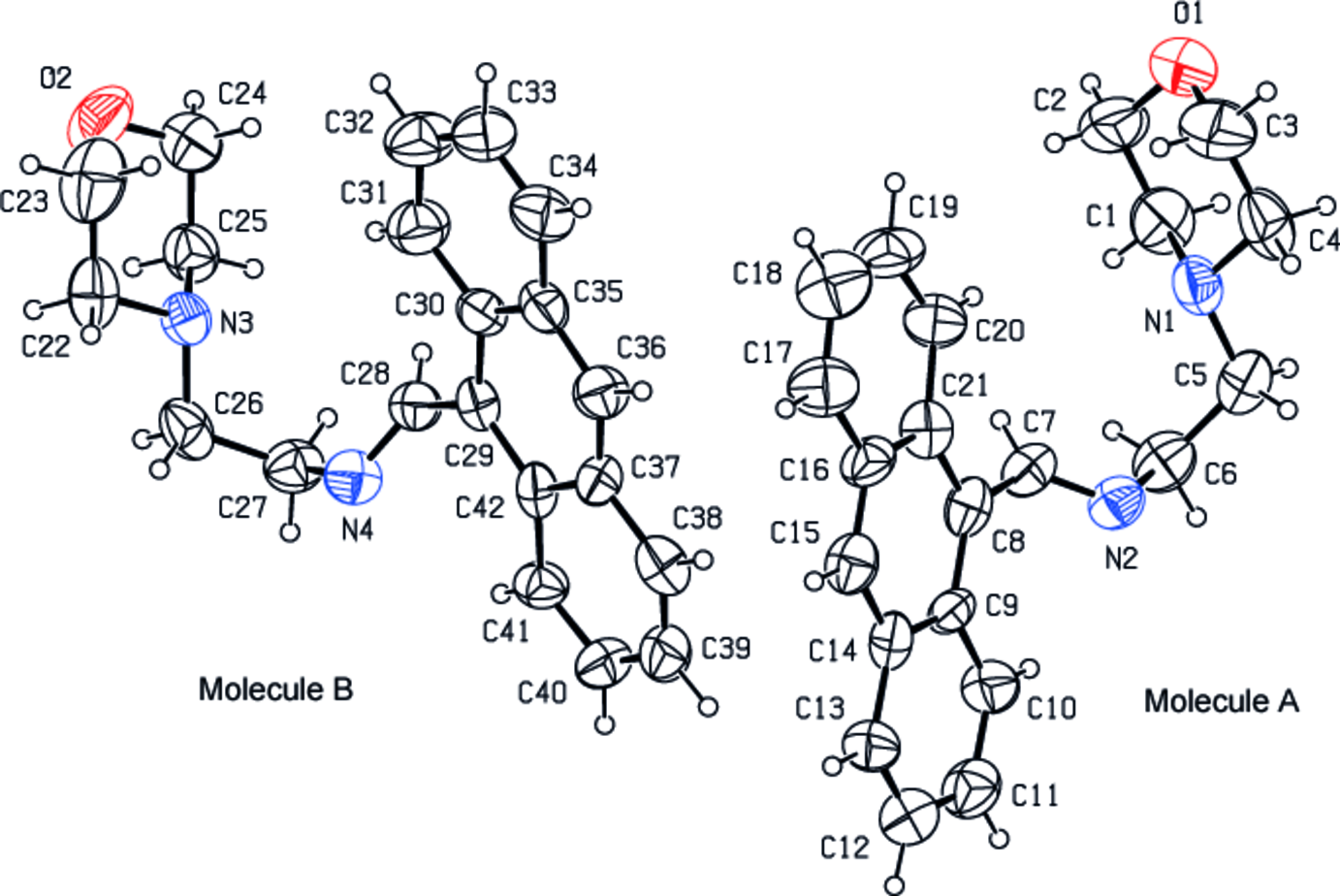

Supplement: Supplementary file 4 [file e-70-o1045-fig1.tif]

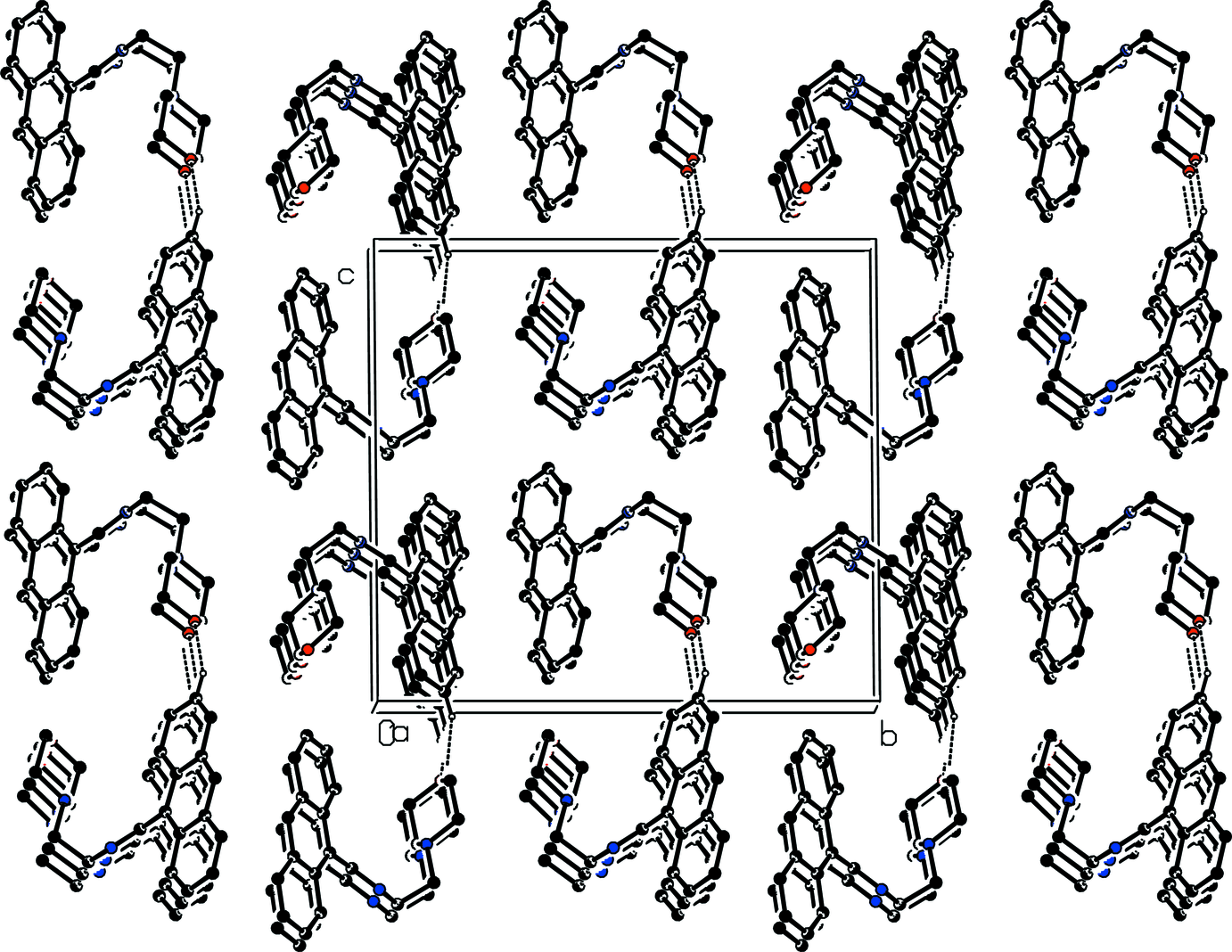

Supplement: Supplementary file 5 [file e-70-o1045-fig2.tif]
